# Deterioration of cognitive function after transient cerebral ischemia with amyloid-β infusion—possible amelioration of cognitive function by AT_2_ receptor activation

**DOI:** 10.1186/s12974-020-01775-8

**Published:** 2020-04-07

**Authors:** Li-Juan Min, Jun Iwanami, Masachika Shudou, Hui-Yu Bai, Bao-Shuai Shan, Akinori Higaki, Masaki Mogi, Masatsugu Horiuchi

**Affiliations:** 1 Department of Molecular Cardiovascular Biology and Pharmacology, Ehime University, Graduate School of Medicine, Shitsukawa, Tohon, Ehime 791-0295 Japan; 2 Division of Analytical Bio-Medicine, Advanced Research Support Center (ADRES), Ehime University, Graduate School of Medicine, Tohon, Ehime 791-0295 Japan; 3 Department of Pharmacology, Ehime University, Graduate School of Medicine, Tohon, Ehime 791-0295 Japan

**Keywords:** Cerebrovascular damage, Cerebral ischemia, Amyloid-β peptide, Cognitive impairment, Neuroinflammation, Neuronal degeneration, AT_2_ receptor activation, Cerebral protection

## Abstract

**Background:**

To promote understanding of the pathogenesis of cognitive impairment or dementia, we explored the potential interaction between transient cerebral ischemia and amyloid-β (Aβ) infusion in mediating cognitive decline and examined the possible ameliorative effect of angiotensin II type 2 (AT_2_) receptor activation in vascular smooth muscle cells (VSMC) on this cognitive deficit.

**Methods:**

Adult male wild-type mice (WT) and mice with VSMC-specific AT_2_ receptor overexpression (smAT_2_) were subjected to intracerebroventricular (ICV) injection of Aβ1-40. Transient cerebral ischemia was induced by 15 min of bilateral common carotid artery occlusion (BCCAO) 24 h after Aβ injection.

**Results:**

Aβ injection in WT induced a cognitive decline, whereas BCCAO did not cause a significant cognitive deficit. In contrast, WT with BCCAO following Aβ injection exhibited more marked cognitive decline compared to Aβ injection alone, in concert with increases in superoxide anion production, nicotinamide adenine dinucleotide phosphate (NADPH) oxidase activity, and expression of p22phox, p40phox, monocyte chemoattractant protein (MCP)-1 and interleukin (IL)-1β in the hippocampus, and upregulation of RAGE (receptor for advanced glycation end product), an Aβ transporter. BCCAO following Aβ injection further enhanced neuronal pyknosis in the hippocampus, compared with BCCAO or Aβ injection alone. In contrast, smAT_2_ did not show a cognitive decline, increase in oxidative stress, inflammation, and RAGE level or neuronal pyknosis, which were induced by BCCAO with/without Aβ injection in WT.

**Conclusions:**

Transient cerebral ischemia might worsen Aβ infusion-mediated cognitive decline and vice versa, with possible involvement of amplified oxidative stress and inflammation and impairment of the RAGE-mediated Aβ clearance system, contributing to exaggerated neuronal degeneration. AT_2_ receptor activation in VSMC could play an inhibitory role in this cognitive deficit.

## Introduction

Cognitive impairment and dementia are becoming serious health problems globally that impair the quality of life of peoples at all ages; hence, promising strategies for prevention and inhibition of their onset and development are urgently needed [1]. Currently, the leading cause of cognitive impairment or dementia is not fully understood. An excess of amyloid-β (Aβ) peptide in the brain is well recognized to play a pivotal role in the pathogenesis of Alzheimer’s disease (AD)-related dementia by triggering multiple events such as enhancement of oxidative stress, activation of microglia and astrocytes, and neuroinflammation [2]. Moreover, both clinical and animal studies have demonstrated that excessive Aβ could induce cerebrovascular pathologies, such as cerebrovascular atherosclerosis and cerebral amyloid angiopathy (CAA), not only leading to the onset and exacerbation of vascular cognitive impairment or vascular dementia, but also accelerating the development of AD-related dementia [3–6].

In addition to excessive Aβ peptide, it has been highlighted that various vascular risk factors associated with cerebrovascular damage could promote the development of cognitive impairment or dementia, such as ischemia [7]. Supportive evidence from both experimental and clinical studies indicates that transient cerebral ischemia can result in progressive learning and memory deficits, associated with cerebral inflammation and neuronal damage or death contributing to vascular cognitive impairment or vascular dementia [8, 9]. Recently, the relation of ischemic brain damage to AD pathological changes, contributing to the onset and progression of cognitive decline, has emerged. For example, enhanced amyloid precursor protein (APP) expression and Aβ peptide level, or other Alzheimer protein expression, have been demonstrated in the ischemic brain, which caused the development of neuronal degeneration and ultimately cognitive decline of Alzheimer type [10, 11]. In addition, cerebral ischemia has been suggested to elevate brain Aβ level by impairing Aβ clearance system contributing to AD pathological changes [6]. Thus, these findings support the notion that cerebral ischemia might promote the progression of AD pathological features and worsen cognitive deficit in AD.

Therefore, the above collective evidence has revived interest in the idea that cerebrovascular damage and AD pathological features, such as excessive Aβ peptide, are inextricably linked to the expression of cognitive impairment or dementia [12]. Excessive Aβ peptide induces cerebrovascular disorders and aggravates cerebrovascular insufficiency, thereby enhancing neuronal degeneration associated with cerebrovascular damage. On the other hand, cerebrovascular damage causes excessive Aβ peptide generation, possibly through additional impairment of Aβ clearance system, thus resulting in the exaggeration of neuronal degeneration associated with AD [6, 12]. Although individually, these pathways are capable of inducing cognitive decline, their interaction is proposed to enhance their pathophysiological effects on cognition. An increasing evidence has strongly suggested the possible pathological interaction between ischemic brain damage and AD. Some animal studies reported that ischemic-reperfusion brain damage induced Alzheimer’s gene or protein expression contributing to the pathological elevation of Aβ peptide in the brain and development of AD [13, 14]. These Alzheimer proteins in turn might render the brain more susceptible to ischemic pathology and in consequence develop post-ischemic cognitive impairment or dementia. For example, an increased level of soluble Aβ peptide has been reported to render ischemic neurons more vulnerable to apoptosis, excitotoxicity and new ischemic episodes [15–18]. Indeed, the incidence of ischemic stroke is showed to be increased in AD patients [19]. Thus, it is suggested that such altered ischemically brain might further amplify Aβ overproduction contributing to post-ischemic amyloidogenesis and finally full cognitive decline of ischemia with Alzheimer type in a vicious cycle. Therefore, these evidences indicate that there is a close interaction between cerebral ischemia and Aβ peptide leading to cognitive impairment or dementia. Moreover, clinical investigations have established an interaction between cerebral ischemia and AD [20]. However, the more detailed relationship between cerebrovascular damage and Aβ excess, such as whether they have additive or synergistic effects on cognitive decline and the related precise mechanisms, remains to be elucidated.

Accumulating evidence suggests that beyond as a cardiovascular risk factor, angiotensin (Ang) II, a major player in the renin-angiotensin system (RAS) may contribute to the pathogenesis of central nervous system (CNS) disorders via its type 1 (AT_1_) receptor [21]. Our recent findings and some animal and clinical studies have demonstrated that AT_1_ receptor blockers (ARBs) can reduce cerebral ischemic damage [22, 23], improve cognitive decline, and may be associated with a reduced risk of dementia in subjects with or without AD [24–26]. However, the preventive effect of ARBs on cognitive impairment and dementia is still controversial. Stimulation of the Ang II type 2 (AT_2_) receptor, which is widely expressed in fetal tissue, but downregulated rapidly after birth and re-expressed under pathological conditions has recently been expected to exert a cerebral protective effect besides of its cardiovascular protection [27]. AT_2_ receptor activation is suggested to protect against cerebral ischemic damage with/without AT_1_ receptor blockade with an ARB [28–30]. Recently, using AT_2_ receptor-deficient mice or a newly developed AT_2_ receptor agonist, compound 21 (C21), we and others have reported that direct AT_2_ receptor stimulation independent of AT_1_ receptor blockade ameliorated cognitive decline in cerebral ischemia model [31], AD, [32] or vascular dementia mouse model [33]. Therefore, it is conceivable that AT_2_ receptor activation with/without ARB treatment might be more suitable for the prevention and treatment of cerebral damage and is further expected to ameliorate cognitive deficit occurring in more complex conditions.

Therefore, to promote understanding of the pathogenesis of cognitive impairment or dementia, the present study was undertaken to examine the potential interaction between cerebrovascular damage and Aβ excess linked to cognitive decline. Cerebrovascular damage was induced by an operation, bilateral common carotid artery occlusion (BCCAO) that produced transient global cerebral ischemia. In recent studies focusing on the deteriorated role of a vascular risk factor, such as hypertension or transient cerebral ischemia in cognitive function in AD, an experimental model of hypertension (Ang II infusion) or transient cerebral ischemia (BCCAO) constructed in AD (5XFAD mouse) was extensively employed [8, 34]. Moreover, clinical studies have demonstrated that ischemic lesions in combination with Aβ load are critical in generating cognitive deficit [20]. Aspects of AD can be mimicked by intracerebroventricular (ICV) infusion of Aβ in the rodent brain, and this model has been used as an AD mouse model well by many researchers [35]. Recent experimental work investigated insulin signaling in neuronal pathologies in a combined animal model of ischemia elicited by endothelin-1 injection with Aβ ICV infusion [36]. Therefore, in the present study, we established a combined mouse model of BCCAO with Aβ ICV infusion to clarify the hypothesis that transient cerebral ischemia could worsen Aβ infusion-induced cognitive decline and vice versa. To explore new therapeutic approaches to improve cognitive impairment or dementia, we examined the possible ameliorative role of AT_2_ receptor activation in cognition in our mouse model by employing mice with AT_2_ receptor overexpression in vascular smooth muscle cells (VSMC).

## Methods

### Study design, randomization, and blinding

**Fig. 1:**
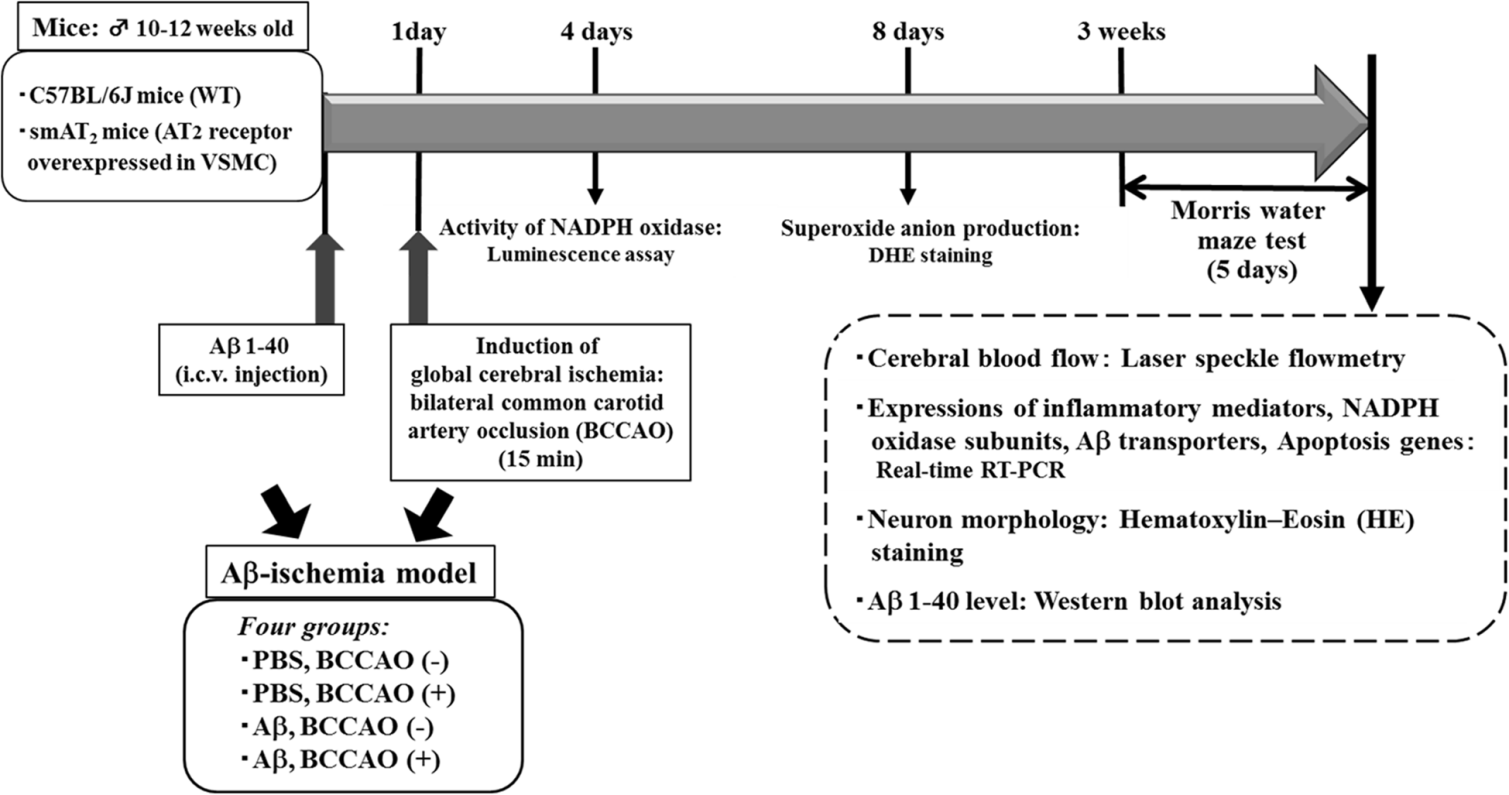
Experimental design

This was a single-institution, randomized, and double-blind controlled study (blinded for investigators and outcomes assessor). All procedures were performed in accordance with the National Institutes of Health guidelines for the use of experimental animals. The experimental protocol was reviewed and approved by the Animal Studies Committee of Ehime University. Per experimental subgroup, the selection of animals was randomized. All animals completed the study, except for the mice in which ICV injection was improper, and no animals were replaced. The sample size of minimal 5 mice per experimental group was chosen based on a formal calculation of power. An overview of the study design is shown in Fig. 1. The number of animals per group was described in each figure legend. No animals were excluded from statistical analysis.

### Animals

Adult (10–12 weeks old) male wild-type (WT) mice (C57BL/6J mice, CLEA Japan Inc., Tokyo, Japan) and mice with AT_2_ receptor overexpression in VSMC (smAT_2_ mice, based on C57BL/6J strain bred in our laboratory) were used in this study. smAT_2_ mice were constructed as reported previously [37] and obtained from the Kyoto Prefectural Medical University. And these transgenic mice were confirmed as VSMC-specific AT_2_ receptor overexpressing mice. There may be a sex difference in AT_2_ receptor re-expression in pathologic conditions, and the sex difference in AT_2_ receptor re-expression under our experimental conditions could cause sex differences in cognitive function. Moreover, the sex difference in estrogen expression might also influence cognitive function, although the results of estrogen on the behavior tests are still unclear. Therefore, to exclude the possible influence of sex differences in AT_2_ receptor re-expression and estrogen, we used only male mice in the present study. Moreover, in order to rule out the influence of aging on the association of cerebral ischemia combined with Aβ infusion with cognitive decline, we chose the adult mice aged at 10–12 weeks old to construct Aβ-ischemia mouse model, since aging is the greatest risk factor for most diseases including neurodegenerative diseases and cognitive impairment. These animals were housed in a room in which lighting was controlled (12 h on, 12 h off) and the room temperature was maintained at 25 °C. They were given a standard diet (MF, Oriental Yeast Co., Ltd., Tokyo, Japan) and water ad libitum.

### Experimental protocol

To examine the possible interaction between cerebrovascular dysfunction and Aβ excess in mediating cognitive decline, we constructed an Aβ-cerebral ischemia mouse model. As shown in Fig. 1, we infused mice with Aβ1-40 by ICV injection. After 24 h, to induce cerebrovascular damage, transient global cerebral ischemia was induced by carrying out bilateral common carotid artery occlusion (BCCAO) for 15 min. Mice were weighed before and 1 week and 2 weeks after Aβ1-40 injection with/without BCCAO, and immediately before each analysis, and showed no significant change in body weight at each time point in each group between smAT_2_ and WT mice. Three weeks after Aβ1-40 injection with/without BCCAO, cognitive function was evaluated. After the cognitive task, cerebral blood flow (CBF) was analyzed, and after that, brain samples were obtained and used for various analyses. We did not observe a significant difference in blood pressure in each group between two strains 3 weeks after Aβ1-40 injection. To examine NADPH oxidase activity and superoxide anion production, brain samples were acquired 4 days and 8 days after Aβ1-40 injection, respectively.

### Preparation of Aβ peptide

Aβ1-40 was purchased from Peptide Institute (Osaka, Japan) and was prepared as a stock solution at a concentration of 66.7 pmol/ul in sterilized PBS (0.54 mg Aβ1-40 peptide was dissolved in 1.9 ml sterilized PBS), and aliquots were stoked at – 20 °C. Aβ1-40 solution was aggregated by incubation at 37 °C for 2 days before use. The conversion degree of Aβ1-40 solution after incubation was confirmed by transmission electron microscopy (TEM) as described previously [38]. The Aβ1-40 solution was placed on a copper collodion carbon-coated grid (carbon deposition with vapor deposition equipment (JEE-420: JEM Led)). After 5~10 min adsorption at room temperature, samples were counterstained with 1% uranyl acetate (MERCK Ltd., Germany) and then examined under a JEM 1230 (JEOL Ltd., Japan) at 80 kV. Images (4 k × 2.7 k:11 million pixels) were captured with a side-entry Orius 1000 TEM CCD camera (model no 785, Gatan Led, USA).

### ICV injection of Aβ1-40

We produced an AD mouse model mimicked by ICV injection of Aβ1-40 in the mouse brain, because this model has been commonly used as an animal model of AD, which are validated by the reliability in detail based on three criteria (face validity, construct validity, and predictive validity) [35]. ICV injection was performed as described previously [39]. Mice were randomly divided into Aβ1-40 infusion group and vehicle control group. Each mouse was anesthetized with sodium pentobarbital (50 mg/kg intraperitoneal) and fixed in a stereotactic frame. The skull was sterilized by using gauze embedded in 75% ethanol and opened with a cranial drill. A volume of 200 pmol/3 μl of Aβ1-40 was injected slowly into the lateral ventricle unilaterally (1 mm to right of midline, 0.2 mm posterior to bregma, and 2.5 mm ventral to skull surface) at a rate of 1 μl/min. For injection, a 28-gauge stainless-steel needle connected by polyethylene tubing to a microsyringe (25 ul, Hamilton) with a microsyringe pump (KD Scientific, MA, USA) was used. After injection, the needle was held in the original location for an additional 5 min and then withdrawn. For vehicle control mice, 3 μl PBS was injected. Body temperature was maintained with a heat lamp throughout the procedure and recovery. After they were completely alert, mice were returned to their homeroom, and normal food and water were given. General locomotor activity and diet volume of mice were checked daily and did not change significantly in each mouse until sample preparation. The accurate placement of the injection (needle track) was confirmed during the dissection of the mice to conduct biochemistry assays. Results from mice presenting cannula misplacement or any sign of cerebral hemorrhage were discarded from the group (overall less than 5% of the total mice used). To verify that injection was carried out properly and stably, we also used another group of mice to be subjected to ICV injection of the same volume of India ink. The distribution of the injected ink throughout both sides of the lateral ventricles was confirmed in more than 90% of mice.

### Transient global cerebral ischemia induced by BCCAO

One day after ICV injection, mice in Aβ1-40 infusion group or vehicle control group were randomly selected to receive an operation of transient global cerebral ischemia or sham operation with anesthesia, after the mice presenting cannula misplacement or any sign of cerebral hemorrhage with ICV injection were discarded. Transient global cerebral ischemia was induced by BCCAO as described previously [40]. Briefly, the bilateral common carotid arteries were isolated and gently occluded with microvascular clips through an anterior cervical incision. After 15 min, the clips were removed to allow reperfusion. Control mice were sham-operated without occlusion. The postoperative care received by mice following BCCAO was similar to that for mice following Aβ1-40 injection. Cerebral ischemia level was detected by an apparent unilateral decrease of CBF and/or apparent color change of the brain. There was no significant difference in CBF during BCCAO in each mouse.

### Morris water maze test (MWMT)

Spatial learning memory as a measure of cognitive function of mice at 3 weeks after injection of Aβ1-40 or PBS was evaluated by MWMT as described previously [24]. All mice selected for ICV injection plus BCCAO or sham operation were subjected to MWMT. Each treated mouse was trained 5 times a day at 20-min intervals for 5 consecutive days. In each trial, mice were given 120 s to find the platform. Swimming was video-tracked (AnyMaze, Wood Dale, IL, USA), and latency, path length, swimming speed, and cumulative distance from the platform were recorded. Mean escape latency and mean swimming speed for all trials on each day in each group was calculated.

### Measurement of CBF

After MWMT, CBF was determined by laser speckle flowmetry (Omegazone, laser speckle blood flow imager, Omegawave, Tokyo, Japan) as described previously [29]. Briefly, a midline incision was made in the scalp of the mice with anesthesia. A 780-nm laser semiconductor laser illuminated the whole skull surface. Mean CBF was measured on the skull surface. Light intensity was accumulated in a charge-coupled device camera and transferred to a computer for analysis. Image pixels were analyzed to produce average perfusion values.

### Western blot analysis

Total protein was prepared from the mouse hippocampus. The proteins were subjected to SDS-PAGE and immunoblotted with anti-β-amyloid (D54D2) XP® Rabbit antibody (Cell Signaling Technology, Inc., Beverly, Mass) or anti-β-actin antibody (Sigma-Aldrich, Inc., St. Louis, MO). Visualization of proteins and densitometric analysis were performed as described previously [24].

### Detection of superoxide anion production

Superoxide anion production in the hippocampus was detected as described previously [24]. In brief, frozen, enzymatically intact, 10-μm-thick sections of the brain were stained with dihydroethidium (DHE: 10 μM, Sigma-Aldrich, Inc., St. Louis, MO) in phosphate-buffered saline (PBS) for 30 min at 37 °C in a humidified chamber protected from the light. For the detection of ethidium, samples were examined with an Axioskop microscope (Axioskop 2 plus with AxioCam, Carl Zeiss, Oberkochen, Germany) equipped with a computer-based imaging system. The intensity of the fluorescence was analyzed using a computer-imaging software (Densitograph, ATTO Corp., Tokyo, Japan), normalized as fluorescence intensity per area, and expressed as fold change relative to the value of PBS, BCCAO (−) group.

### NADPH oxidase activity

The activity of NADPH oxidase in the total protein of the hippocampus homogenate was measured with a luminescence assay as previously described [41, 42], using 500 μM lucigenin as the electron acceptor and 100 μM NADPH as the substrate. The reaction was started by the addition of 80 μg protein to the lucigenin and NADPH mixture. Samples were incubated for 5 min at room temperature. Chemiluminescence was monitored with a luminometer (AB-2200, ATTO Corp., Tokyo, Japan).

### Real-time quantitative reverse-transcription polymerase chain reaction (RT-PCR)

After CBF measurement, total RNA was extracted from the hippocampus with Sepasol reagent (Nacalai Tesque, Inc., Kyoto, Japan). Real-time quantitative RT-PCR was performed using SYBR Premix Ex Taq (Takara Bio Inc., Japan). The level of target gene expression was normalized against expression of a housekeeping gene, glyceraldehyde-3-phosphate dehydrogenase (GADPH), in each sample. PCR primers were as follows: monocyte chemotactic and activating factor-1 (MCP-1), 5′-TTAACGCCCCACTCACCTGCTG-3′ (forward) and 5′-GCTTCTTTGGGACACCTGCTGC-3′ (reverse); tumor necrosis factor-α (TNF-α), 5′-CGAGTGACAAGCCTGTAGCC-3′ (forward) and 5′-GGTGAGGAGCACGTAGTCG-3′ (reverse); interleukin-6 (IL-6), 5′-CCACTTCACAAGTCGGAGGCTTA-3′ (forward) and 5′-GCAAGTGCATCATCGTTGTTCATAC-3′ (reverse); interleukin-4 (IL-4), 5′-TCGGCATTTTGAACGAGGTC-3′ (forward) and 5′-GAAAAGCCCGAAAGAGTCTC-3′ (reverse); interleukin-1β (IL-1β), 5′-TCCAGGATGAGGACATGAGCAC-3′ (forward) and 5′-GAACGTCACACACCAGCAGGTTA-3′ (reverse); p47phox, 5′-GTCCCTGCATCCTATCTGGA-3′ (forward) and 5′-GGGACATCTCGTCCTCTTCA-3′ (reverse); p40phox, 5′-TTTGAGCAGCTTCCAGACGA-3′ (forward) and 5′-GGTGAAAGGGCTGTTCTTGC-3′ (reverse); p22phox, 5′-TGGCTACTGCTGGACGTTTCAC-3′ (forward) and 5′-CTCCAGGAGACAGATGAGCACAC-3′ (reverse); p67phox, 5′-CAGACCCAAAACCCCAGAAA-3′ (forward) and 5′-AGGGTGAATCCGAAGCTCAA-3′ (reverse); gp91phox, 5′-TGGGATCACAGGAATTGTCA-3′ (forward) and 5′-CTTCCAAACTCTCCGCAGTC-3′ (reverse); SOD1, 5′-GAGACCTGGGCAATGTGACT-3′ (forward) and 5′-GTTTACTGCGCAATCCCAAT-3′ (reverse); SOD2, 5′-CCGAGGAGAAGTACCACGAG-3′ (forward) and 5′-GCTTGATAGCCTCCAGCAAC-3′ (reverse); SOD3, 5′-ATCCCACAAGCCCCTAGTCT-3′ (forward) and 5′-GTGCTATGGGGACAGGAAGA-3′ (reverse); LRP1, 5′-GACCAGGTGTTGGACACAGATG-3′ (forward) and 5′-AGTCGTTGTCTCCGTCACACTTC-3′ (reverse); RAGE, 5′-TGACCGCAGTGTAAAGAGTCCC-3′ (forward) and 5′-CCCTTAGCTGGCACTTAGATGG-3′ (reverse); caspase-3, 5′-GCAAGATTTGGCGATACTATGA-3′ (forward) and 5′-TTCCAGCCTTTGACTCTGCT-3′ (reverse); Bax, 5′-GAGATGAACTGGACAGCAATATGG-3′ (forward) and 5′-GCAAAGTAGAAGAGGGCAACCA-3′ (reverse); Bcl-2, 5′-GTACCTGAACCGGCATCTG-3′ (forward) and 5′-GGGGCCATATAGTTCCACAA-3′ (reverse); VEGF-A, 5′-CACGACAGAAGGAGAGCAGAAGT-3′ (forward) and 5′-TTCGCTGGTAGACATCCATGAA-3′ (reverse); collagen IV, 5′-CAAGCATAGTGGTCCGAGTC-3′ (forward) and 5′-AGGCAGGTCAAGTTCTAGCG-3′ (reverse); GAPDH, 5′-TGCGACTTCAACAGCAACTC-3′ (forward) and 5′-ATGTAGGCCATGAGGTCCAC-3′ (reverse).

### Histological analysis

Following MWMT, mice chosen randomly from each group were anesthetized and then perfused transcardially with ice-cold saline followed by 4% paraformaldehyde. The brain was removed and then immersion-fixed in the same fixative at 4 °C, dehydrated, and then embedded in paraffin blocks. Coronal and 5-μm-thick paraffin serial slices were cut on a microtome. Coronal slices chosen between 1.82 and 2.31 mm posterior to the bregma for hippocampal observation were selected in a systematic-uniform-random manner (8–10 total) for hematoxylin-eosin (HE) staining. The histomorphologic appearance of neurons was visualized with an Axioskop 2 microscope (Carl Zeiss, Oberkochen, Germany) at × 400 magnification equipped with a computer-based imaging system, and the cells in the dentate gyrus (DG) were set as the observation range. The average cell numbers of pyknotic neurons and total neurons per field from 2 slices with a 200-μm interval along the whole length of the hippocampus in each coronal slice were counted respectively with a computer-imaging software (Densitograph, ATTO Corp., Tokyo, Japan).

### Statistical analysis

The mouse genotype and/or all treatments were not considered as relevant factors for the statistical analysis. All values are expressed as mean ± SEM. Data were analyzed with *F* test followed by Student’s or Welch’s *t* test to assess the difference between two groups. For multiple comparison analyses, one-way ANOVA method was used, then, when significant differences were indicated, the post hoc pairwise comparison was performed using Tukey-Kramer method to adjust for multiple hypothesis testing. For each experiment, the difference with a value of *p* < 0.05 between groups was considered statistically significant.

## Results

### Molecular properties of Aβ1-40

After incubation at 37 °C for 2 days, to investigate the molecular properties of Aβ1-40 injected in mice, we performed TEM. Supplemental Figure S1 shows the particle species contained in the aggregated Aβ1-40 solution. Throughout the microscope grid, we observed that the Aβ1-40 solution was composed of a mixture of more the soluble oligomers and less the insoluble fibrils. Only parts of the aggregation exhibited long and straight fibrils with some intertwining fibrils.

### Cognitive function

**Fig. 2:**
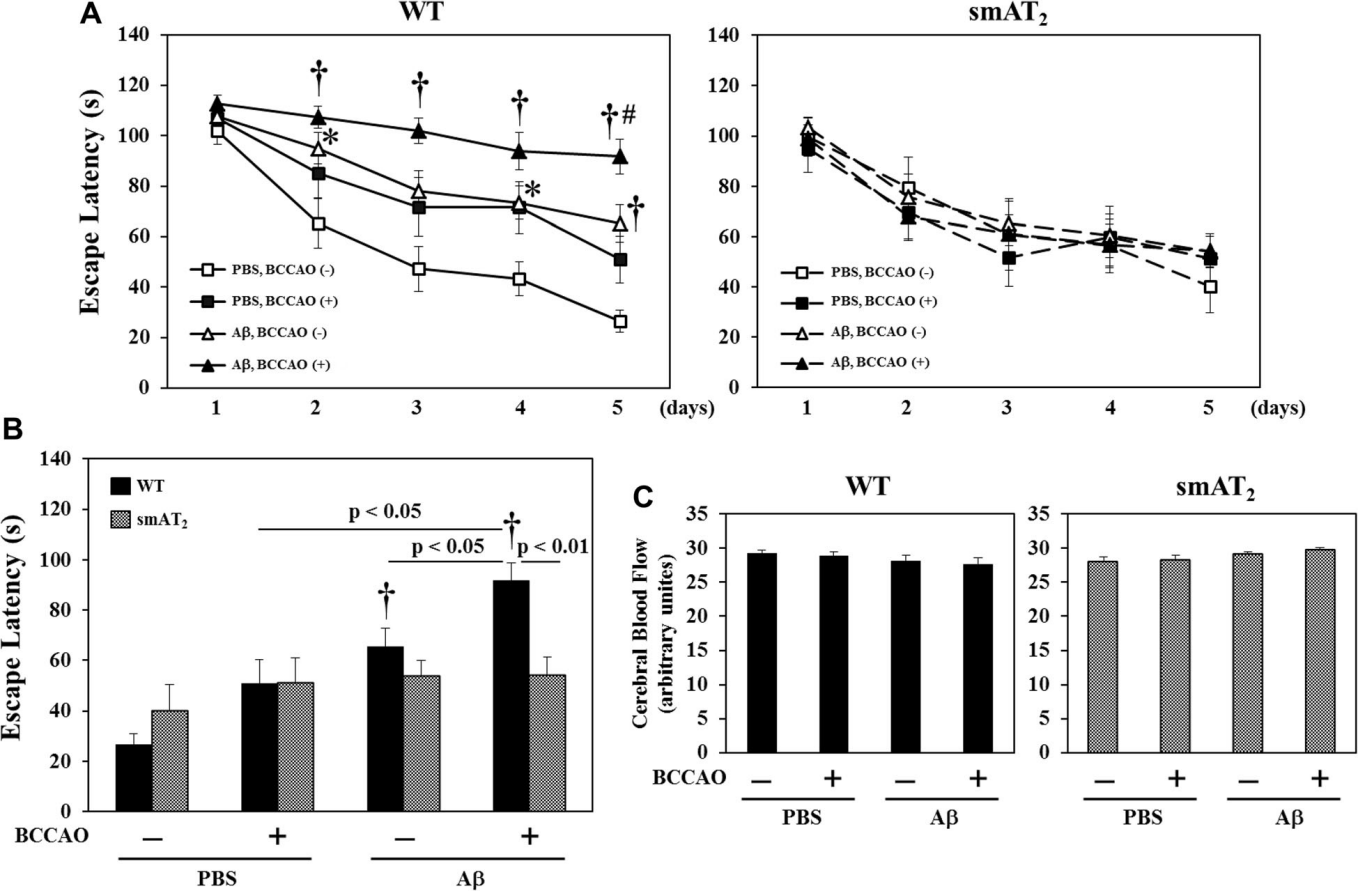
Effect of BCCAO with/without Aβ1-40 on cognitive function evaluated by MWMT. Three weeks after injection, MWMT was performed. Time courses of escape latency from day 1 to day 5 in MWMT in WT and smAT_2_ (**a**) mice are shown. **b** Comparison of escape latency on day 5 between WT and smAT_2_ mice. Comparison of CBF is shown in **c.** After the MWMT, CBF was determined by laser speckle flowmetry. Quantification of the volume of mean CBF analyzed by computer software is shown. ^*^P < 0.05, ^†^*P* < 0.01 vs. PBS, BCCAO (−) in WT on days 2, 3, 4, and 5, respectively. ^#^*P* < 0.05 vs. PBS, BCCAO (+) and Aβ, BCCAO (−) in WT on day 5, respectively. *n* = 12 in WT-PBS, BCCAO (−); *n* = 12 in WT-PBS, BCCAO (+); *n* = 15 in WT-Aβ, BCCAO (−); *n* = 14 in WT-Aβ, BCCAO (+); *n* = 8 in smAT_2_-PBS, BCCAO (−); *n* = 9 in smAT_2_-PBS, BCCAO (+); *n* = 14 in smAT_2_-Aβ, BCCAO (−); *n* = 14 in smAT_2_-Aβ, BCCAO (+)

In WT strain, compared with mice with PBS injection without BCCAO, mice with Aβ injection without BCCAO exhibited longer escape latency, whereas mice with BCCAO following PBS injection tended to show lower spatial learning and memory, but the difference was not significant. Interestingly, escape latency of mice with BCCAO following Aβ injection was much longer than that of mice with Aβ injection without BCCAO or mice with BCCAO following PBS injection (Fig. 2a), suggesting that mice with BCCAO following Aβ injection undergo significantly more marked cognitive decline compared with mice with Aβ injection without BCCAO or mice with BCCAO following PBS injection. On the other hand, in smAT_2_ strain, there were no significant differences in escape latency among all groups (Fig. 2a). Figure 2b shows that on day 5 of MWMT, the cognitive function of mice without Aβ without BCCAO treatment did not differ significantly between WT and smAT_2_ strains. smAT_2_ mice with BCCAO following Aβ injection did not show significantly impaired cognitive function compared with WT mice with the same treatment, demonstrating that AT_2_ receptor signaling prevents the enhanced cognitive decline by BCCAO combined with Aβ injection. We observed that there was no significant difference in mean swimming speed in each group in both WT and smAT_2_ mice, and mean swimming speed was similar between two strains, suggesting that the result of spatial learning and memory was not significantly interfered by the change in swimming ability (Supplemental Figure S2). The change in cerebral blood flow (CBF) after the MWMT was compared in each group. Both in WT and smAT_2_ mice, no significant difference in mean CBF was observed among all groups (Fig. 2c).

### Oxidative stress markers

**Fig. 3:**
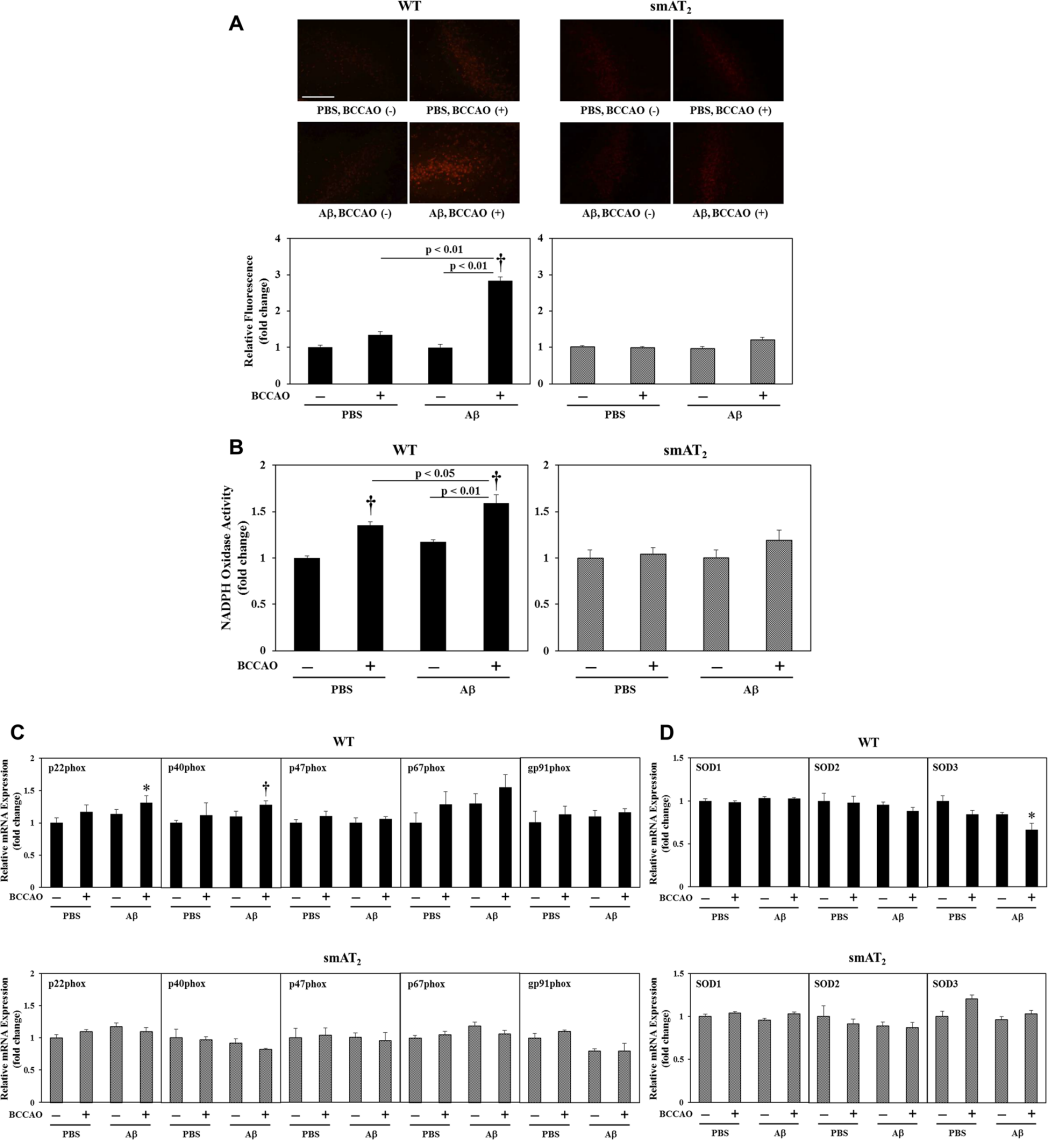
Effect of BCCAO with/without Aβ1-40 on oxidative stress markers. **a** Superoxide anion production in the hippocampus 8 days after injection determined by DHE staining. Representative photographs in each group were from the same location in the DG area, × 200 magnification. Scale bar = 250 μm. *n* = 6 for each group in WT and smAT_2_, respectively. **b** NADPH oxidase activity in the hippocampus 4 days after injection determined by luminescence assay. Expressions of NADPH oxidase subunits (**c**) and superoxide dismutase (SOD) (**d**) in the hippocampus 3 weeks after injection were determined by real-time RT-PCR. *n* = 9 for each group in WT; *n* = 6 for each group in smAT_2_. ^*^P < 0.05, ^†^*P* < 0.01 vs. PBS, BCCAO (−), respectively

In WT strain, mice with BCCAO following PBS injection and mice with Aβ injection without BCCAO did not show a significant increase in superoxide anion production in the hippocampus, whereas mice with BCCAO following Aβ injection exhibited marked enhancement of superoxide anion production, compared with that in mice with BCCAO or Aβ injection alone (Fig. 3a). Figure 3b shows that in WT mice, nicotinamide adenine dinucleotide phosphate (NADPH) oxidase activity in the hippocampus was increased in mice with BCCAO following PBS injection, and this observed increase was further enhanced in mice with BCCAO following Aβ injection. According to the results of mRNA expression in the hippocampus, compared with WT mice with PBS injection without BCCAO, expression of NADPH oxidase subunit p22phox and p40phox in WT mice with BCCAO following Aβ injection was significantly increased (Fig. 3c), and expression of SOD3 was significantly decreased (Fig. 3d), although no significant changes of these markers were observed in mice with BCCAO or Aβ injection alone. On the other hand, in smAT_2_ strain, no significant differences were observed in these oxidative stress markers among all groups (Fig. 3a–d).

### Expression of inflammatory mediators

**Fig. 4:**
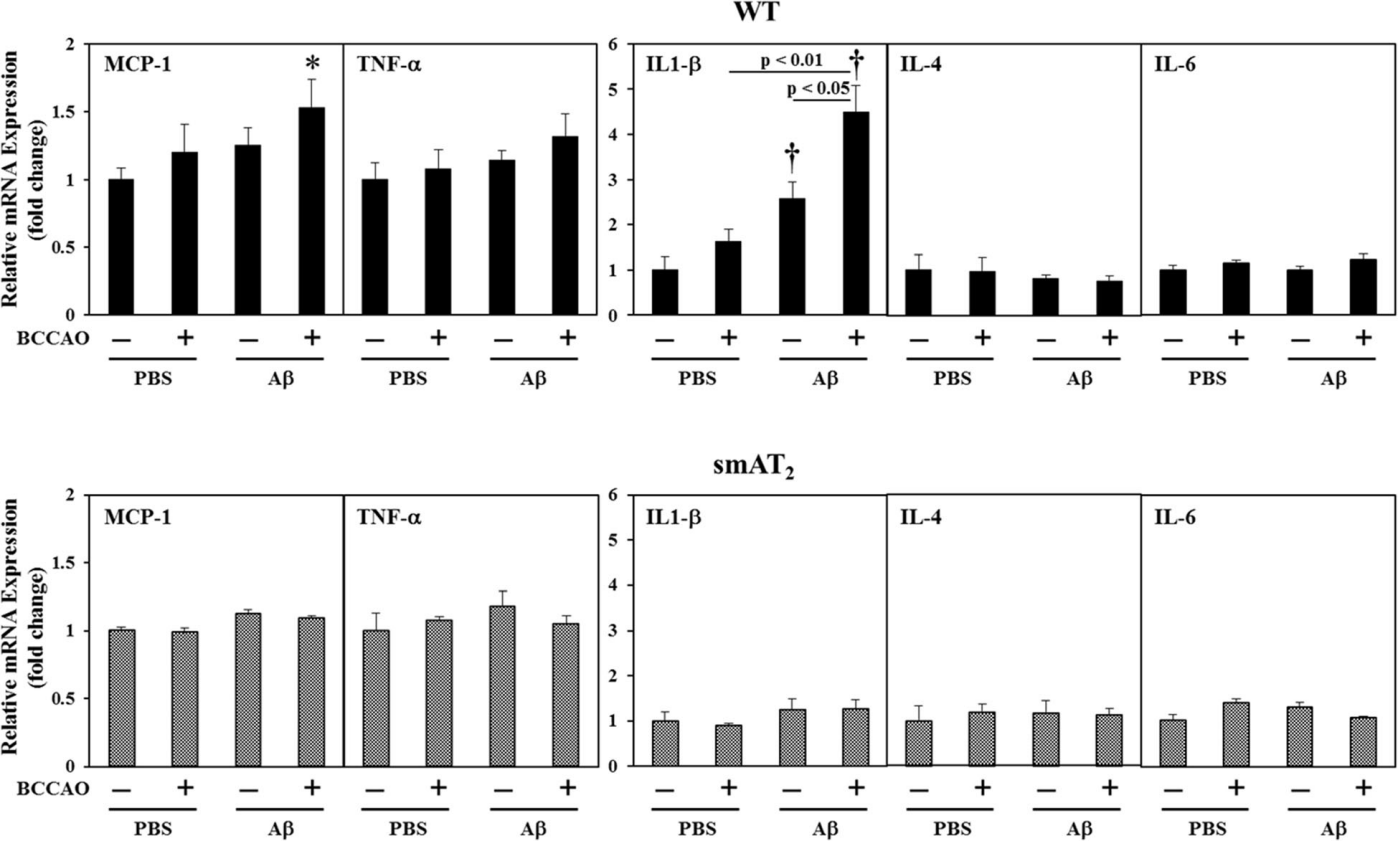
Effect of BCCAO with/without Aβ1-40 on inflammatory markers. mRNA expressions of inflammatory mediators in the hippocampus 3 weeks after injection were determined by real-time RT-PCR in WT and smAT_2_ mice. ^*^P < 0.05, ^†^*P* < 0.01 vs. PBS, BCCAO (−), respectively. *n* = 9 for each group in WT; *n* = 6 for each group in smAT_2_

In WT strain, compared with mice with PBS injection without BCCAO, mice with BCCAO following PBS injection and mice with Aβ injection without BCCAO did not show a significant increment in expression of inflammatory mediators such as monocyte chemoattractant protein (MCP)-1 in the hippocampus, whereas mice with BCCAO following Aβ injection exhibited a significant increase in MCP-1 expression. Strikingly, interleukin (IL)-1β expression was significantly increased in mice with Aβ injection without BCCAO, and this increase was further strengthened in mice with BCCAO following Aβ injection. On the other hand, no significant discrepancy in these inflammatory mediators among all groups was observed in smAT_2_ mice (Fig. 4).

### Expression of Aβ transporters

**Fig. 5:**
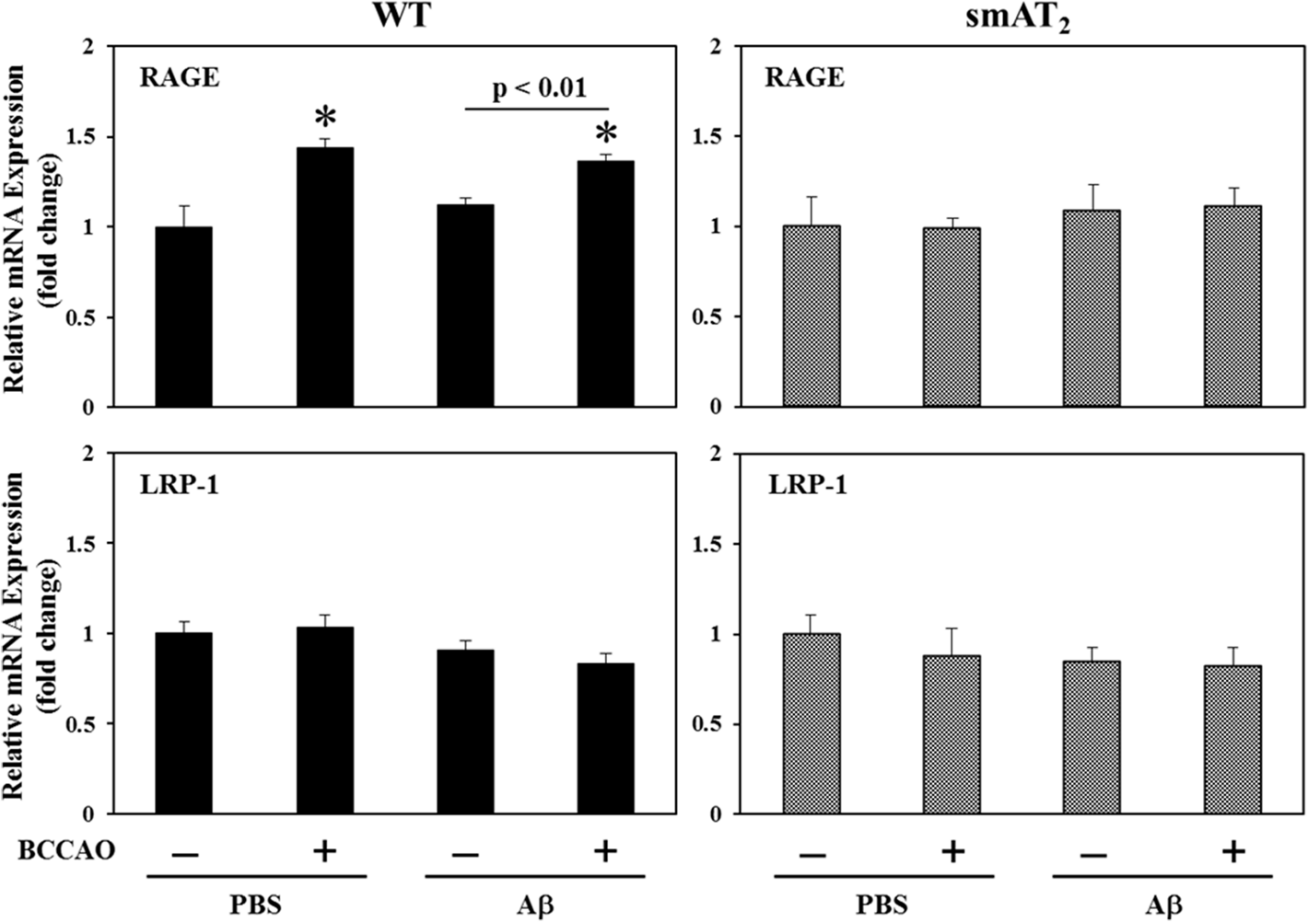
Effect of BCCAO with/without Aβ1-40 on Aβ transporters. mRNA expressions of RAGE and LRP-1 in the hippocampus 3 weeks after injection by real-time RT-PCR in WT and smAT_2_ mice are shown. ^*^P < 0.05 vs. PBS, BCCAO (−). *n* = 6 for each group in WT and smAT_2,_ respectively

Receptor for advanced glycation end products (RAGE) and low-density lipoprotein receptor-related protein-1 (LRP-1) are suggested to may act as Aβ transporters and mediate Aβ clearance system to regulate brain Aβ level [43, 44]. We next examined mRNA expression of RAGE and LRP-1. In WT strain, compared with mice with PBS injection without BCCAO, mice with BCCAO following PBS injection exhibited a significant increase in RAGE expression in the hippocampus. Although RAGE expression was not changed in mice with Aβ injection without BCCAO, it was also enhanced significantly in mice with BCCAO following Aβ injection. However, LRP-1 expression was similar in all groups. On the other hand, there was no significant difference in both RAGE and LRP-1 expression among all groups in smAT_2_ mice (Fig. 5).

### Neuronal degeneration

**Fig. 6:**
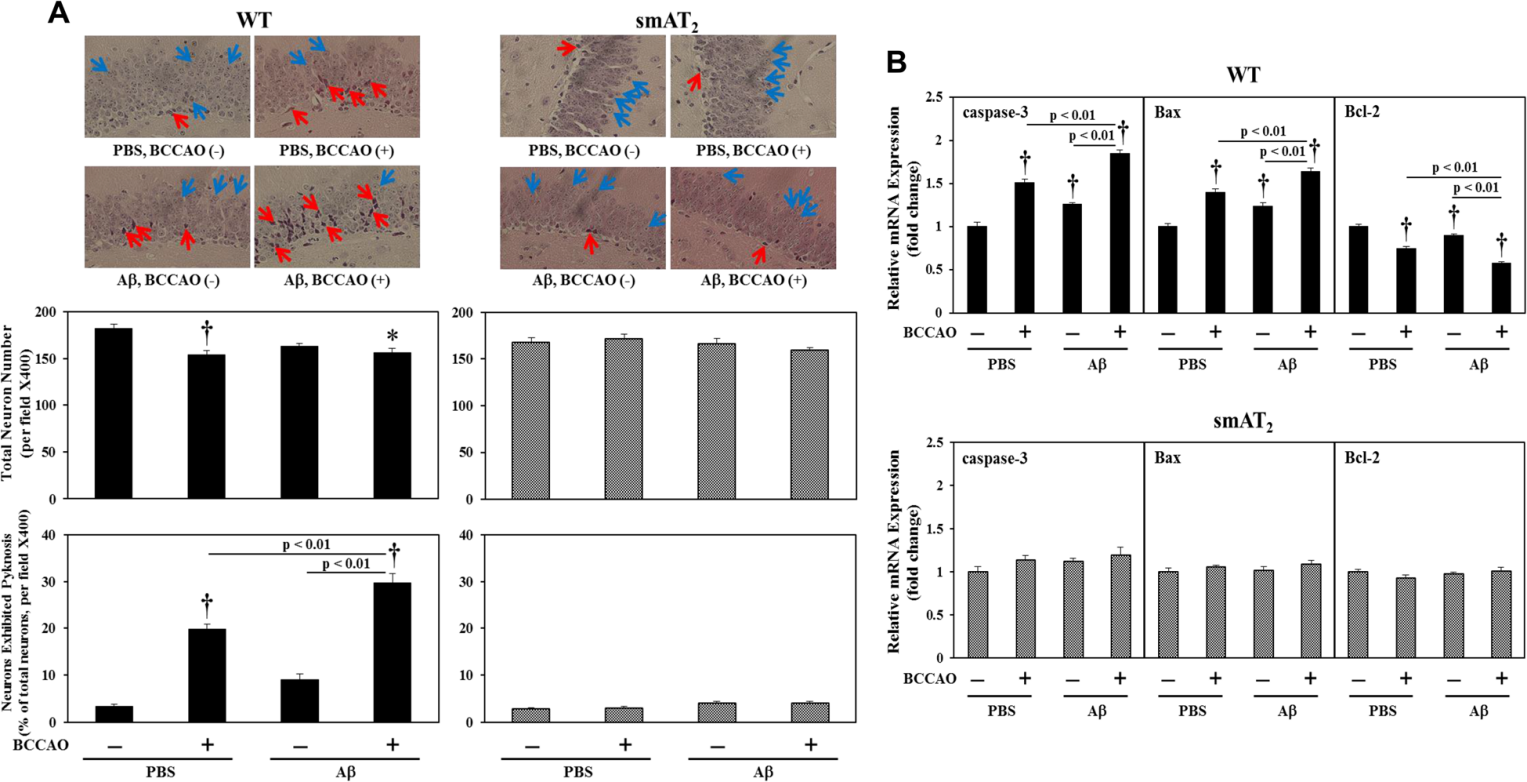
Effect of BCCAO with/without Aβ1-40 on neuronal degeneration. **a** Neuronal degeneration in the hippocampus 3 weeks after injection evaluated by histological analysis with HE staining. Representative photographs in each group were from the same location in the DG area, × 400 magnification. Neurons with rich cytoplasm and round and slightly stained nucleus with relatively large and clear nucleolus formation indicate normal cells (blue arrow). Neurons shrunken with condensed and deeply stained nucleus indicate pyknotic cells (red arrow). Quantification of total neuronal cell number and percentage of pyknotic neurons calculated and analyzed at × 400 are shown. *n* = 5 for each group in WT and smAT_2,_ respectively. **b** Expressions of apoptosis genes in the hippocampus 3 weeks after injection by real-time RT-PCR. *n* = 6 for each group in WT and smAT_2,_ respectively. ^*^P < 0.05, ^†^*P* < 0.01 vs. PBS, BCCAO (−), respectively

Neuronal pyknosis detected by HE staining has widely been used as a marker for neuronal degeneration after ischemic brain damage recently [45]. To evaluate neuronal degeneration, we examined neuronal pyknosis with HE staining and analyzed mRNA expression of apoptosis genes. HE staining of the hippocampal neurons revealed normal cells with rich cytoplasm and round and slightly stained nucleus with relatively large and clear nucleolus formation. In contrast, degenerated neurons exhibited atrophic cytoplasm and shrunken bodies. Most degenerated neurons were shrunken with a condensed and deeply stained nucleus, and some of them showed irregular morphology with small cell soma, which was defined as neuronal pyknosis. In WT strain, compared with mice with PBS injection without BCCAO, in mice with BCCAO following PBS injection, most neurons in the hippocampal dentate gyrus (DG) region exhibited pyknosis, and the number of total neurons (normal cells and pyknotic cells) was significantly decreased. Although neuronal pyknosis and total neuronal cell number were not changed in mice with Aβ injection without BCCAO, pyknotic cells were further enhanced in mice with BCCAO following Aβ injection compared with that in mice with BCCAO following PBS injection (Fig. 6a). Moreover, real-time RT-PCR of apoptosis genes showed that in WT strain, in mice with BCCAO following PBS injection and mice with Aβ injection without BCCAO, hippocampal expressions of caspase-3 and Bax were enhanced, but Bcl-2 expression was reduced compared with those in mice with PBS injection without BCCAO. And these effects on apoptosis gene expression were further significantly strengthened by BCCAO following Aβ injection (Fig. 6b). On the other hand, there were no significant differences in neuronal pyknosis and total neuronal cell number, and apoptosis gene expression among all groups in smAT_2_ mice (Fig. 6a, b). We also observed that there was no significant difference in the basal levels of both neuronal pyknosis and total neuronal cell number between WT and smAT_2_ mice (Fig. 6a).

### Aβ1-40 protein level

**Fig. 7:**
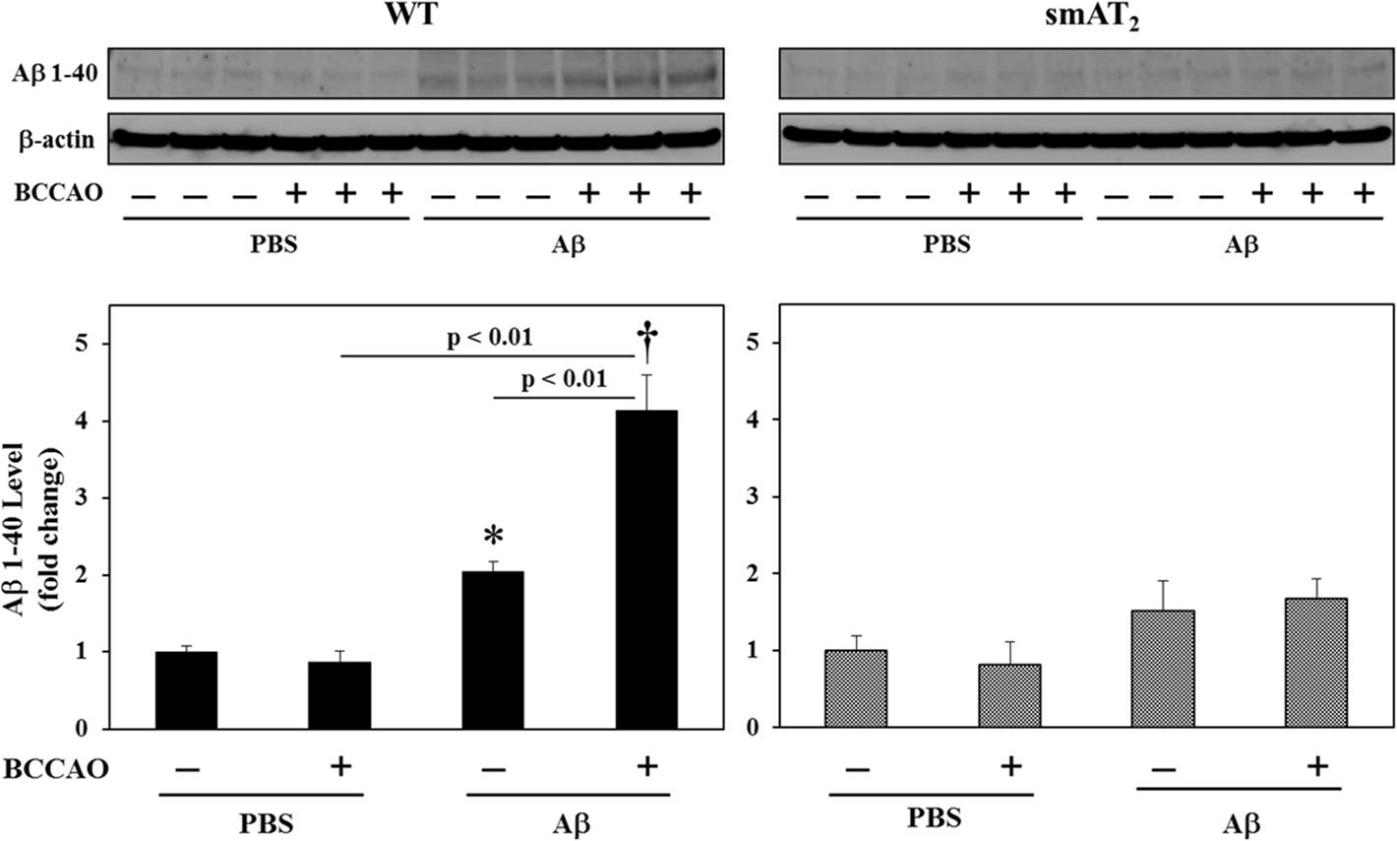
Effect of BCCAO with/without Aβ1-40 on Aβ1-40 protein level. Aβ1-40 level in the hippocampus 3 weeks after injection was examined by western blot analysis. Representative photographs are shown from different experiments. The bands of each group are shown from 3 different mice, respectively. The mean densitometric measurements of the bands were normalized by β-actin, and the data were expressed as fold change relative to the value of PBS, BCCAO (−) group. *P < 0.05, ^†^*P* < 0.01 vs. PBS, BCCAO (−), respectively. *n* = 6 for each group in WT and smAT_2,_ respectively

Aβ1-40 protein level in the mouse hippocampus was examined by western blot analysis. In WT strain, Aβ1-40 level was increased in mice with Aβ injection without BCCAO, compared with mice with PBS injection without BCCAO. Interestingly, this enhancement was further elevated in mice with BCCAO following Aβ injection. In contrast, in smAT_2_ mice, there was no significant difference in Aβ1-40 level among all groups (Fig. 7).

## Discussion

A strong relationship is implicated between cerebrovascular damage and Aβ excess in mediating cognitive impairment or dementia [12]. The findings of our present study are consistent with this notion. Using the MWMT, which is the most popular behavioral assay for detecting AD-related cognitive decline, we demonstrated here that transient cerebral ischemia could exacerbate Aβ infusion-induced cognitive decline and vice versa, because WT mice with BCCAO combined with Aβ injection exhibited more marked cognitive decline compared to mice with Aβ injection alone or BCCAO alone. In other words, transient cerebral ischemia and Aβ infusion at least exert an additive effect in mediating cognitive decline. Our results indicated that cerebrovascular damage mechanisms might contribute to a faster and exacerbated development of neuropathology and cognitive deficit in our mouse model, since BCCAO alone significantly downregulated hippocampal expressions of cerebrovascular function markers, collagen IV, and vascular endothelial growth factor A (VEGF-A) in WT mice, whereas Aβ injection alone had no effects on them. Interestingly, these downregulated expressions of collagen IV and VEGF-A were further reduced after BCCAO with Aβ infusion (Supplemental Figure S3).

Although there is evidence suggesting an ameliorative effect of ARBs on cognitive decline associated with diabetes, AD, or dementia [24–26], large clinical trials have shown no significant difference in the incidence of dementia between treatment with an ARB and the placebo group, and the detailed reason why ARBs failed to prevent dementia is not well understood. In contrast, our recent data showed that direct AT_2_ receptor stimulation with C21 independent of ARB treatment significantly improved cognitive decline in AD [32] or vascular dementia mouse model [33]. In the present study, mice with AT_2_ receptor overexpression in VSMC, smAT_2_ mice exhibited similar cognitive function to WT mice, and Aβ injection with/without BCCAO did not result in a significant decline in cognitive performance with no cerebrovascular alterations in smAT_2_ mice (Supplemental Figure S3), suggesting that AT_2_ receptor activation might ameliorate cerebrovascular damage and further cognitive decline in our mouse model. Therefore, despite lack of AT_1_ receptor blockade with an ARB, AT_2_ receptor activation in the cerebrovasculature might be beneficial for the prevention and treatment of cognitive decline caused by transient cerebral ischemia with Aβ infusion, besides of its classical vascular protection.

Although the exact cellular and molecular mechanisms remain elusive and difficult to verify, oxidative stress and inflammation associated with cerebral ischemia or excessive Aβ are proposed to contribute to cognitive decline. Nicotinamide adenine dinucleotide phosphate (NADPH) oxidase activity and superoxide levels in the ischemic brain showed an early increase and peaked 2 h after reperfusion in a transient focal ischemia rat model [46], while oxidative stress markers were reported to upregulate 1 day–10 days after Aβ injection [47, 48]. We expected to observe the additive or synergistic enhancements of oxidative stress markers by cerebral ischemia and Aβ infusion, but no or small alterations by each treatment alone; therefore, we examined NADPH oxidase activity at 4 days and superoxide anion production at 8 days after injection. In fact, we observed that BCCAO and Aβ injection additively caused a significant increase in NADPH oxidase activity. Moreover, BCCAO and Aβ injection synergistically induced enhancement of superoxide anion production, accompanied by increases in NADPH oxidase subunits, p22phox and p40phox mRNA expression and a decrease in an antioxidant enzyme, superoxide dismutase (SOD) 3 mRNA expression. Therefore, data from the present study support the possibility that transient cerebral ischemia with Aβ infusion produced superoxide anions through an increase in expression and phosphorylation of cytoplasmic p40phox and enhanced binding to membrane p22phox, thereby contributing to the accumulation of oxidative stress. Thus, our findings suggested that transient cerebral ischemia interacts with Aβ infusion to worsen cognitive function through the enhancement of oxidative stress by increasing NADPH oxidase activity and p22phox and p40phox mRNA expression, and lowering the SOD3-mediated antioxidant defense. However, the link between ROS-induced oxidative DNA damage and neuronal damage or death evaluated by 8-OHdG level in our combined mouse model needs to be investigated in the future.

The most studied inflammatory mediators in the ischemic brain or Aβ neurotoxicity are the cytokines interleukin (IL)-1, IL-6, IL-10, and ΤΝF-α, and the chemokine MCP-1. Following a 20-min transient global cerebral ischemia in rats, IL-1β mRNA and protein expression were increased not only during early reperfusion (1 h), but also at later times (6–24 h), indicating biphasic expression [49]. Moreover, in rats with transient MCAO, MCP-1 level in the ischemic hippocampus was upregulated from 6 h, peaked at 2 days and thereafter gradually decreased [50]. In an AD rat model with Aβ1-40 injection, the hippocampus showed significantly increased mRNA and protein expression of IL-1β 16 days after injection [51] and presented upregulation of other inflammatory mediators after 21 days [52]. Moreover, ICV injection of Aβ1-42 to the mouse induced marked production of IL-1β, ΙL-6, and MCP-1 in the hippocampus with little production of TNF-α [53]. These reports implicated that ischemia induces inflammatory mediator expression at an early phase after reperfusion, while Aβ injection increases inflammatory mediator expression after a very long time. We expected to observe the additive or synergistic increases in inflammatory mediator expression by cerebral ischemia and Aβ infusion, but no or small alterations by each treatment alone; therefore, we analyzed mRNA expression of all inflammatory mediators 3 weeks after Aβ injection. Accordingly, it was difficult to obtain detectable changes for various inflammatory mediators after BCCAO or Aβ infusion alone after such a long period, except for IL-1β because of its long persistence time. Very interestingly, BCCAO following Aβ injection resulted in significant increases in mRNA expression of MCP-1 and IL1-β compared with each treatment alone or control, but other mediators including TNF-α expression had no change. These data suggested that transient cerebral ischemia and Aβ infusion at least additively upregulate the level of key inflammatory mediators, MCP-1 and IL1-β even after a long period, thereby contributing to exacerbated the cognitive decline.

In cerebral ischemia, ROS generation initiated by activation of the NADPH oxidase pathway has been demonstrated to stimulate ischemic cells to secrete inflammatory mediators in the cerebrovasculature and peripheral leukocyte recruitment, then activated inflammatory cells can release more cytokines and more ROS and other cytotoxic agents [54]. The Aβ peptide in the brain itself can induce a local inflammatory response through microglial and astrocytic activation [55]. Conversely, inflammatory mediators, such as IL-1β has been shown to increase amyloid precursor protein (APP) production in astrocytes leading to Aβ upregulation in the brain and impaired neuronal function [56]. Moreover, some studies using MCP-1-deficient mice indicate that MCP-1 signaling-mediated IL-1β upregulation influences BBB disruption and aggravates ischemia-related neuronal damage [57]. Therefore, these findings lead to a speculation that all the processes including the occurrence of transient cerebral ischemia or Aβ excess, amplification of oxidative stress, and MCP-1/IL-1β-mediated inflammation might create a vicious cycle in mediating cognitive decline.

Accumulating evidence has highlighted the important role of Aβ clearance system in regulating brain Aβ level [44]. Cerebral clearance of soluble Aβ involves both degradation in the brain and brain-to-blood elimination across the BBB [58]. It has been suggested that Aβ transporters in vascular cells can transfer Aβ across the BBB and thus mediated clearance of Aβ from the brain; alternatively, Aβ transporters may also mediate Aβ clearance via phagocytosis of Aβ by microglia and astrocytes [59]. Both the receptor for advanced glycation end products (RAGE) and low-density lipoprotein receptor-related protein (LRP)-1 which are expressed in a range of cells including endothelial cells, VSMC, neurons, and glial cells in the brain may act as Aβ transporters and are proposed to associate with Aβ clearance [43, 44]. Moreover, RAGE may interact with its ligand including Aβ to mediate multiple cellular events involving generation of ROS and induction of inflammatory response associated with vascular damage and neuronal degeneration, and its expression can be upregulated by ROS and cytokines [40, 60]. Brain expression of RAGE has been shown to be increased in AD mouse models and in AD patients relating to excessive Aβ [61, 62], whereas the expression of LRP-1 is decreased in AD brain [63]. Recently, the effect of RAGE expression on ischemic stroke has been studied. Kamide et al. reported that in mice with BCCAO, RAGE expression in the hippocampus was induced starting at 12 h and maintained until 7 days compared with control mice. They also demonstrated that RAGE plays a critical role in the initial vascular damage and subsequent glia-mediated inflammation and delayed neuronal death after BCCAO [40]. Thus, we assumed that RAGE could be an important molecule linking transient cerebral ischemia and Aβ infusion in inducing the exacerbation of cognitive decline. Accordingly, we observed that BCCAO alone induced a significant increase in RAGE expression in the hippocampus. Although RAGE expression in the brain has been suggested to be substantially increased in an Aβ-rich environment [61, 62], we did not observe an alteration of RAGE expression after Aβ injection alone. This contradiction might be due to our experimental model of exogenous Aβ uptake. However, Aβ injection combined with BCCAO also caused significant enhancement of RAGE expression. On the other hand, expression of LRP-1 after BCCAO with/without Aβ injection showed no significant difference from control. Our findings are consistent with the hypothesis that transient cerebral ischemia can cause impairment of RAGE-mediated Aβ clearance system to accelerate an excess of Aβ in the brain through oxidative stress and MCP-1/IL-1β-mediated vascular inflammation, and amplify Aβ-mediated oxidative stress and glial inflammation, then, overproduction of Aβ peptide and associated amplification of oxidative stress and inflammation promote the development of cerebral ischemia, thereby potentiating ischemia-mediated neuronal damage and finally full cognitive decline of ischemia with Alzheimer type in a vicious cycle. Our results are also consistent with previous observations showing a weak or no association of LRP-1 with Aβ clearance system and AD progression [64]. Therefore, we pointed out a preventive role of RAGE in mediating Aβ1-40 clearance system contributing to transient cerebral ischemia with Aβ infusion deteriorated cognitive function, with the lacking of LRP-1-mediated clearance of Aβ from the brain.

The Aβ1-40 solution we injected into mice was composed of a mixture of more the soluble oligomers and less the insoluble fibrils (Supplemental Figure S1). There are several studies showing no Aβ deposition in the ischemic brain after cerebral ischemia including BCCAO with/without AD [8, 9]. On the other hand, previous studies reported that approximately 1% of the total amount of injected Aβ1-40 reached to the bilateral hippocampus at 1 h, and then, it was slowly cleared in a time-dependent manner, and no fibrillary plaques of Aβ1-40 were observed in ICV injection mouse model [16, 35]. Thus, even fibrillary forms were contained when using Aβ1-40 to ICV inject to the mice, it appears that the evaluation of fibrillary plaques in the brain is quite difficult. Actually, employing Congo red staining and Direct fast scarlet staining, we could not detect fibrillary plaques in WT brain 3 weeks after Aβ1-40 injection with/without BCCAO in either staining method (data not shown). However, employing western blot analysis, we observed that Aβ1-40 protein level in WT hippocampus significantly enhanced after Aβ1-40 injection and BCCAO compared with each treatment alone, indicating that Aβ1-40 level is increased in the brain accompanied by enhanced RAGE expression associating with impairment of Aβ1-40 clearance system. It has been demonstrated that soluble Aβ1-40 oligomer level rather than insoluble fibrillary plaque density correlates with the extent of cognitive decline in AD and the underlying mechanisms of neurotoxicity [35], and an increase in soluble Aβ oligomers in AD brains precedes amyloid plaque formation and correlates with the development of vascular pathology [65]. Therefore, we demonstrated here that in response to cerebral ischemia combined with Aβ1-40 infusion, the level of soluble Aβ1-40 oligomers is elevated in the brain accompanied by enhanced RAGE expression associating with impairment of Aβ1-40 clearance system and mainly contributes to the amplified oxidative stress, glia inflammation, neuronal damage, and final deteriorated cognitive function. However, the involvement of relative APP expression or secretase activity which is associated with Aβ production and secretion routes in our mouse model needs to be further defined.

The cognitive deficit caused by cerebral ischemia or an elevation of Aβ has shown a close association with neuronal damage or death [45, 66]. Apoptosis during cerebral ischemia/reperfusion injury plays a major role in the process of neuronal death [67]. Aβ peptide has been reported to induce neuronal apoptosis in the hippocampus in transgenic mice and also in Aβ-infused mice contributing to neuronal degeneration and cognitive deficit (16). These neuronal apoptoses were mainly mediated by caspase-3-dependent signaling cascade which involves pro-apoptotic member Bax and anti-apoptotic member Bcl-2 [16, 67]. Therefore, we evaluated neuronal degeneration by examining neuronal morphological change with HE staining and expression of apoptosis genes. We observed that BCCAO alone caused significant morphological change with an increase in neuronal pyknosis, with enhanced expressions of caspase-3 and Bax, reduced Bcl-2 expression in the hippocampus 3 weeks after injection. Although Aβ injection alone did not significantly induce morphological characteristics of neuronal degeneration, the increased expressions of caspase-3 and Bax and decreased Bcl-2 expression could be observed in the Aβ injection alone group. And most importantly, the changes of morphological characteristics and apoptosis gene expression by each treatment alone were further markedly enlarged by BCCAO and Aβ injection, indicating the possible contribution of neuronal degeneration in the hippocampus to exacerbated cognitive function induced by cerebral ischemia with Aβ infusion. Our results indicated that the induction of apoptotic cells might be the major cause of the effect of cerebral ischemia with Aβ infusion on neuronal degeneration. Recently, emerging evidence indicates that the autophagic pathway plays a role in cerebral ischemia or Aβ-induced delayed neuronal death [45, 68]. However, whether this pathway is involved in our mouse model is not clear at this time, and future studies to resolve this issue may help us further understanding the mechanisms of neuronal degeneration resulting from the interaction between transient cerebral ischemia with Aβ infusion.

In our mouse model, ischemia/reperfusion has occurred. We have confirmed ischemia level in this model histologically and observed the significant ischemic area in the bilateral cerebral hemispheres in WT mice with BCCAO with/without Aβ injection, whereas the whole brain in the sham mice showed no detectable ischemic area. We have also confirmed ischemia level by evaluating cerebral vascular reactivity through measuring CBF and found that 15 min after BCCAO, CBF significantly decreased to 20~30% of its pre-occlusion value in WT mice, and it gradually recovered after reperfusion and restored nearly to its pre-occlusion value 24 h after reperfusion (data not shown). This pattern of CBF change after BCCAO is similar to the observations reported previously [45]. Accordingly, our results showed that BCCAO with/without Aβ injection did not induce a significant reduction of CBF 3 weeks after Aβ injection, and no obvious difference in CBF was observed among all groups in both two strains. These present data suggested that CBF seems not to participate in the pathological mechanisms underlying transient cerebral ischemia with Aβ infusion-exacerbated cognitive function. However, in our CBF measurement, the skull surface was illuminated diffusely by a laser light; thus, the data of mean CBF indicated mean CBF of the surface of bilateral cerebral hemispheres, and it could not directly refract blood flow of other regions, such as the hippocampus. If the method of CBF measurement could be ameliorated to directly analyze blood flow of vascular territories of hippocampus, it was possible that cerebral ischemia with Aβ infusion might reduce the ability of blood flow recovery after reperfusion, thereby causing blood flow reduction in the WT hippocampus compared with others 3 weeks after injection. Therefore, the method of CBF measurement used at this time might be a limitation for considering the involvement of cerebrovascular alterations. Moreover, it needs to be further confirmed whether Aβ deposits in the cerebral vessel in our combined mouse model.

Compared with WT mice, in smAT_2_ mice, there were no significant changes in NADPH oxidase activity, superoxide anion production, expressions of inflammatory mediators and NADPH oxidase subunits, RAGE and Aβ1-40 level, and neuronal degeneration markers among all groups, indicating that the potential inhibitory effect of AT_2_ receptor activation on the cognitive deficit in our mouse model might be due to attenuation of amplified oxidative stress and inflammation, improvement of RAGE-mediated Aβ clearance system, downregulation of Aβ level, and reduction of neuronal degeneration. Previous reports demonstrated that activation of RAGE in the CNS plays a pivotal role in the cerebral ischemia-induced vascular damage and delayed neuronal death [40]. And recent studies pointed out that specific blocking of RAGE has potential as a future therapeutic approach to decrease the brain uptake of Aβ from the systemic circulation [69]. We proposed here that in response to transient cerebral ischemia combined with Aβ infusion, AT_2_ receptor signaling activated by damaged cerebral vessel could act as a crucial blocker for RAGE expression and activation to prevent Aβ excess in the brain contributing to inhibition of cognitive decline. RAGE is expressed in a range of cells and upregulated by oxidative environment and many cytokines contributing to brain uptake of Aβ [60]. Moreover, our recent studies demonstrated that AT_2_ receptor activation protects against vascular damage and ameliorates cognitive decline possibly by reducing oxidative stress and inflammation [33, 70]. Therefore, we suggested here that AT_2_ receptor activation reduces RAGE expression possibly by attenuating oxidative stress and MCP-1/IL-1β-mediated inflammation in the cerebrovasculature and thus contributing to inhibition of cognitive decline. On the other hand, peroxisome proliferator-activated receptor-gamma (PPAR-γ), a nuclear transcription factor has been suggested to exert neuroprotective effect after cerebral ischemia recently [71]. Moreover, we have reported that direct AT_2_ receptor stimulation by C21 mediated its beneficial effects on vascular proliferation and cerebral ischemic damage through PPAR-γ-mediated pathway and that AT_2_ receptor-interacting protein (ATIP) might be involved in AT_2_ receptor-induced PPAR-γ complex formation to translocate into the nucleus resulting in an attenuation of inflammatory gene expression [70, 72]. Therefore, one of the other mechanisms involved in AT_2_ receptor-downregulated RAGE expression might involve the preventive role of AT_2_ receptor-mediated ATIP-PPAR-γ complex in the transcriptional activity of RAGE.

## Conclusions

Our findings provide animal experimental support indicating that transient cerebral ischemia potentially worsens Aβ infusion-induced cognitive decline and vice versa, and the related cellular and molecular mechanisms might involve enhancement of oxidative stress and inflammation, impairment of RAGE-mediated Aβ clearance system, and exaggeration of neuronal degeneration.

Although additional confirmation is required, we propose here that transient cerebral ischemia may cause cerebrovascular damage and then enhance excessive Aβ in the brain via disruption of RAGE-mediated Aβ clearance system through oxidative stress and inflammation, and Aβ, in turn, may promote the development of cerebral ischemia through amplified oxidative stress and inflammation, thereby contributing ultimately to the full cognitive decline of ischemia with Alzheimer type in a vicious cycle. These findings contribute to the understanding that effective development of therapeutic or preventive strategies targeting Aβ peptide for cognitive impairment or dementia requires consideration of the maintenance of cerebrovascular function.

Our results also imply that AT_2_ receptor activation in the cerebrovasculature could play a preventive role in this worsening of cognitive function through inhibition of oxidative stress, inflammation, impaired RAGE-mediated Aβ clearance system, and neuronal degeneration, thus supporting the notion that targeting AT_2_ receptor activation might be useful for a new generation of therapeutic or preventive strategies targeting Aβ peptide for cognitive impairment or dementia, due to its multiple protective actions both in the vascular system and in the CNS. However, our results need to be further confirmed in clinical trials and translated to clinical value.

## Supplementary information

**Additional file 1: Supplemental Figure S1.** Transmission electron microscopy of Aβ1-40 solution after incubation at 37 °C for two days. Aβ1-40 solution represents a mixture of more the soluble oligomers, monomers and less the insoluble fibrils. Only parts of the aggregation exhibits long and straight fibrils with some intertwining fibrils. Upper photos, X 100,000 magnification. Lower photos, X 200,000 magnification. **Supplemental Figure S2.** Mean swimming speed of Morris Water Maze Test (MWMT). Three weeks after injection, MWMT was performed for 5 days. Time courses of mean swimming speed in each group in WT and smAT_2_ mice are show. n=12 in WT-PBS, BCCAO (-); n=12 in WT-PBS, BCCAO (+); n=15 in WT-Aβ, BCCAO (-); n=14 in WT-Aβ, BCCAO (+); n=8 in smAT_2_-PBS, BCCAO (-); n=9 in smAT_2_-PBS, BCCAO (+); n=14 in smAT_2_-Aβ, BCCAO (-); n=14 in smAT_2_-Aβ, BCCAO (+). **Supplemental Figure S3.** Effect of BCCAO with/without Aβ1-40 on cerebrovascular function markers. mRNA expression of collagen IV and VEGF-A in the hippocampus 3 weeks after injection by real-time RT-PCR in WT and smAT_2_ mice are shown. *P<0.05, ^†^*P*<0.01 vs. PBS, BCCAO (-). n=6 for each group in WT and smAT_2_, respectively.

## Data Availability

Please contact the authors for data requests.

## Abbreviations

Aβ: Amyloid-β
AD: Alzheimer’s disease
Ang II: Angiotensin II
APP: Amyloid precursor protein
AT_1_ receptor: Angiotensin II type 1 receptor
AT_2_ receptor: Angiotensin II type 2 receptor
ARBs: AT_1_ receptor blockers
BCCAO: Bilateral common carotid artery occlusion
CAA: Cerebral amyloid angiopathy
CBF: Cerebral blood flow
CNS: Central nervous system
ICV: Intracerebroventricular
IL-1β: Interleukin-1β
LRP-1: Low-density lipoprotein receptor-related protein-1
MCP-1: Monocyte chemoattractant protein-1
MWMT: Morris water maze test
NADPH oxidase: Nicotinamide adenine dinucleotide phosphate oxidase
RAGE: Receptor for advanced glycation end product
RAS: Renin-angiotensin system
ROS: Reactive oxygen species
smAT_2_: VSMC-Specific AT_2_ receptor overexpression
SOD: Superoxide dismutase
TNF-α: Tumor necrosis factor-α
VSMC: Vascular smooth muscle cells
WT: Wild-type
