# Supplementary material for: Deterioration of cognitive function after transient cerebral ischemia with amyloid-β infusion—possible amelioration of cognitive function by AT2 receptor activation
Source: J Neuroinflammation. 2020 Apr 7;17:106. doi: 10.1186/s12974-020-01775-8 (PMC7140348; doi:10.1186/s12974-020-01775-8)
Supplement: Supplementary file 1 — Additional file 1: Supplemental Figure S1. Transmission electron microscopy of Aβ1-40 solution after incubation at 37 °C for two days. Aβ1-40 solution represents a mixture of more the soluble oligomers, monomers and less the insoluble fibrils. Only parts of the aggregation exhibits long and straight fibrils with some intertwining fibrils. Upper photos, X 100,000 magnification. Lower photos, X 200,000 magnification. Supplemental Figure S2. Mean swimming speed of Morris Water Maze Test (MWMT). Three weeks after injection, MWMT was performed for 5 days. Time courses of mean swimming speed in each group in WT and smAT2 mice are show. n=12 in WT-PBS, BCCAO (-); n=12 in WT-PBS, BCCAO (+); n=15 in WT-Aβ, BCCAO (-); n=14 in WT-Aβ, BCCAO (+); n=8 in smAT2-PBS, BCCAO (-); n=9 in smAT2-PBS, BCCAO (+); n=14 in smAT2-Aβ, BCCAO (-); n=14 in smAT2-Aβ, BCCAO (+). Supplemental Figure S3. Effect of BCCAO with/without Aβ1-40 on cerebrovascular function markers. mRNA expression of collagen IV and VEGF-A in the hippocampus 3 weeks after injection by real-time RT-PCR in WT and smAT2 mice are shown. *P<0.05, †P<0.01 vs. PBS, BCCAO (-). n=6 for each group in WT and smAT2, respectively. [file 12974_2020_1775_MOESM1_ESM.pptx]

## Slide 1
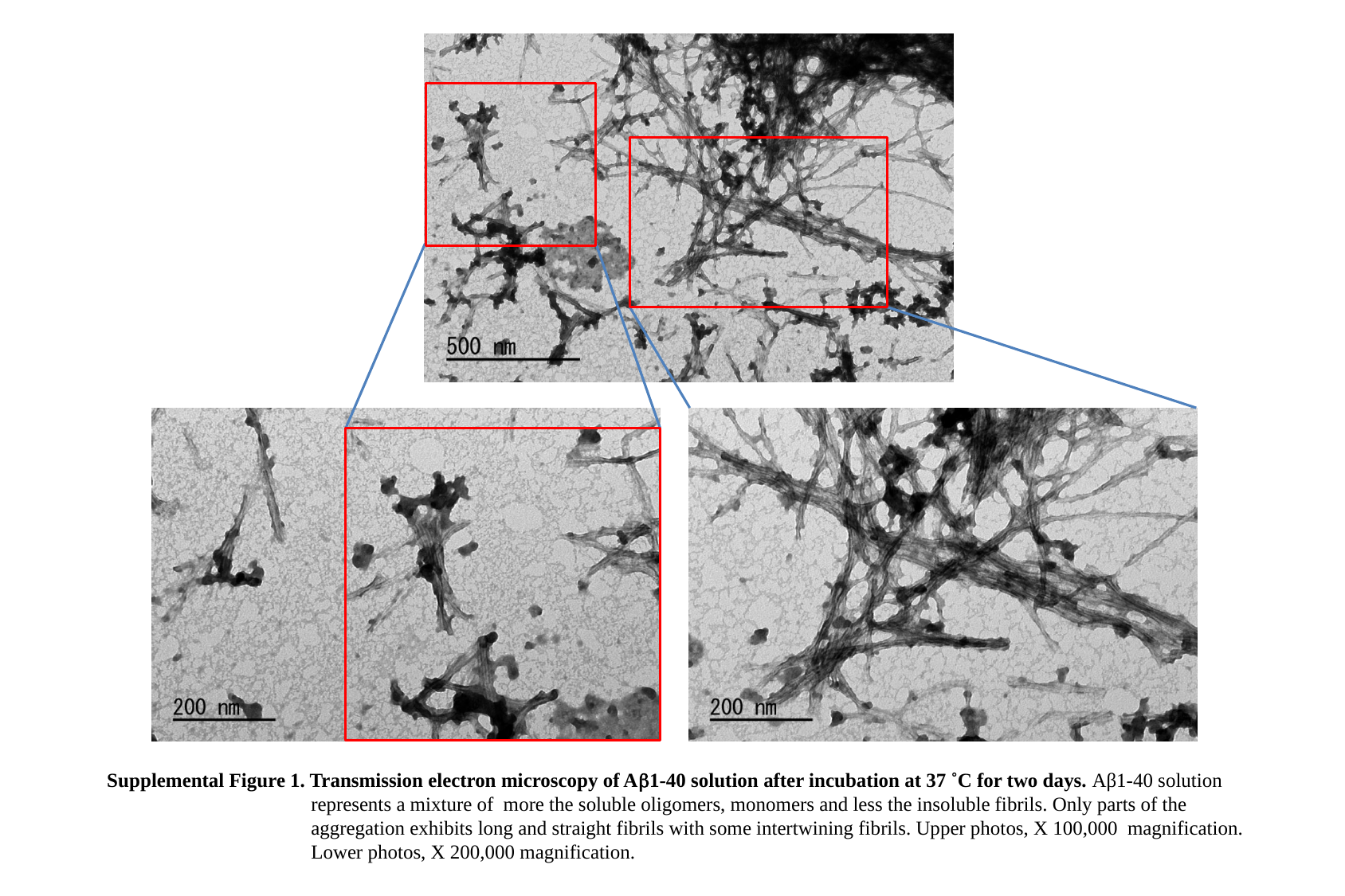

Supplemental Figure 1. Transmission electron microscopy of Ab1-40 solution after incubation at 37 ˚C for two days. Aβ1-40 solution
 represents a mixture of more the soluble oligomers, monomers and less the insoluble fibrils. Only parts of the
 aggregation exhibits long and straight fibrils with some intertwining fibrils. Upper photos, X 100,000 magnification.
 Lower photos, X 200,000 magnification.

## Slide 2
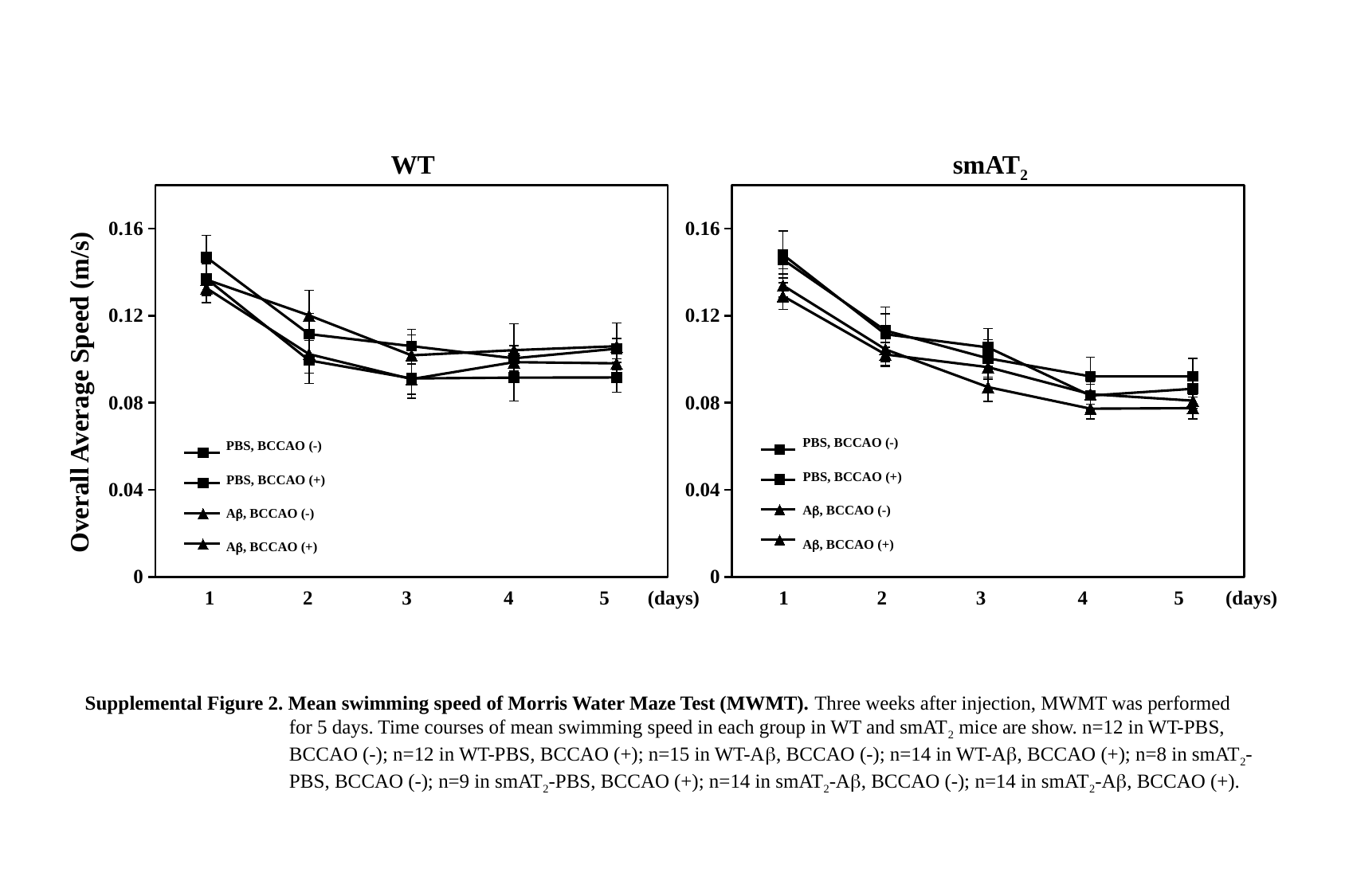

WT
smAT2
### Chart
| Category | PBS, sham | PBS, ischemia | A-beta, sham | A-beta, ischemia |
|---|---|---|---|---|
| day1 | 0.14575 | 0.14802500000000002 | 0.12898333333333337 | 0.13386666666666666 |
| day2 | 0.11315 | 0.11147499999999999 | 0.10219166666666668 | 0.1045 |
| day3 | 0.1004 | 0.10542499999999999 | 0.09635 | 0.08716666666666667 |
| day4 | 0.092125 | 0.08324999999999999 | 0.08392500000000001 | 0.07725000000000001 |
| day5 | 0.092075 | 0.0864 | 0.08093333333333334 | 0.0775 |PBS, BCCAO (-)
PBS, BCCAO (+)
Ab, BCCAO (-)
Ab, BCCAO (+)
### Chart
| Category | PBS, sham | PBS, ischemia | A-beta, sham | A-beta, ischemia |
|---|---|---|---|---|
| day1 | 0.1468 | 0.13696666666666665 | 0.13265333333333335 | 0.13658750000000003 |
| day2 | 0.11155 | 0.09946666666666666 | 0.10235999999999999 | 0.12015 |
| day3 | 0.10601666666666666 | 0.09118333333333334 | 0.09086666666666668 | 0.10178749999999999 |
| day4 | 0.10043333333333333 | 0.09155 | 0.09865333333333333 | 0.10416249999999999 |
| day5 | 0.10483333333333332 | 0.09163333333333333 | 0.09810666666666663 | 0.10597499999999999 |Overall Average Speed (m/s)
PBS, BCCAO (-)
PBS, BCCAO (+)
Ab, BCCAO (-)
Ab, BCCAO (+)
1
2
3
4
5
(days)
1
2
3
4
5
(days)
Supplemental Figure 2. Mean swimming speed of Morris Water Maze Test (MWMT). Three weeks after injection, MWMT was performed
 for 5 days. Time courses of mean swimming speed in each group in WT and smAT2 mice are show. n=12 in WT-PBS,
 BCCAO (-); n=12 in WT-PBS, BCCAO (+); n=15 in WT-Aβ, BCCAO (-); n=14 in WT-Aβ, BCCAO (+); n=8 in smAT2-
 PBS, BCCAO (-); n=9 in smAT2-PBS, BCCAO (+); n=14 in smAT2-Aβ, BCCAO (-); n=14 in smAT2-Aβ, BCCAO (+).

## Slide 3
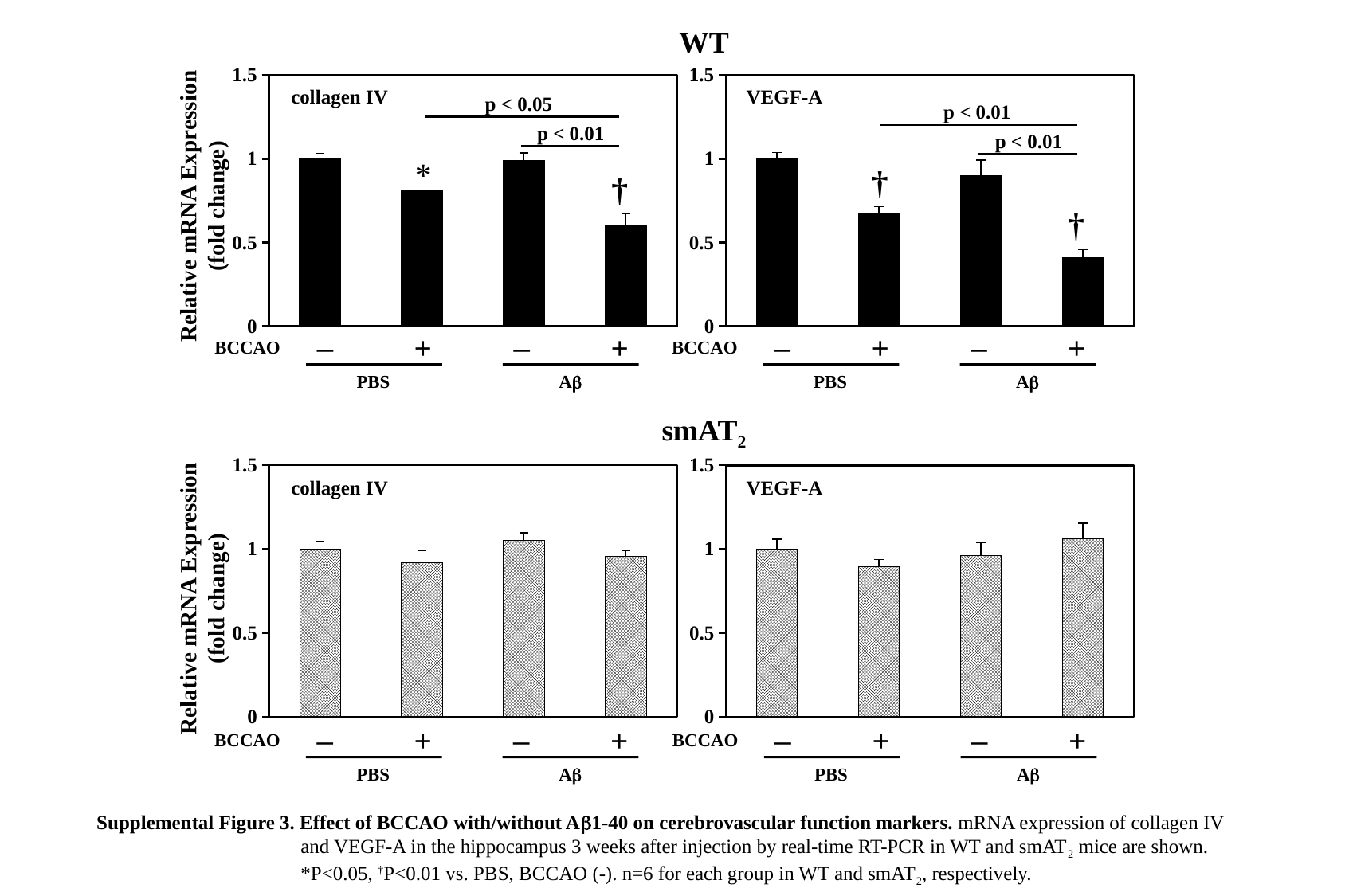

WT
### Chart
| Category | |
|---|---|
| PBS, sham | 0.9999999650864217 |
| PBS, ischemia | 0.8130399540942198 |
| A-beta, sham | 0.9886332099271131 |
| A-beta, ischemia | 0.5990840982737596 |*
### Chart
| Category | |
|---|---|
| PBS, sham | 0.999999978189524 |
| PBS, ischemia | 0.6709949385320869 |
| A-beta, sham | 0.8999184996546523 |
| A-beta, ischemia | 0.4093627086244495 |collagen IV
VEGF-A
p < 0.05
p < 0.01
p < 0.01
p < 0.01
†
†
Relative mRNA Expression
(fold change)
†
–
+
–
+
BCCAO
PBS
Ab
–
+
–
+
BCCAO
PBS
Ab
smAT2
### Chart
| Category | |
|---|---|
| PBS, sham | 1.000000013120106 |
| PBS, ischemia | 0.9185711067026068 |
| A-beta, sham | 1.0511585172326487 |
| A-beta, ischemia | 0.9566218288416821 |
### Chart
| Category | |
|---|---|
| PBS, sham | 1.0000001198891095 |
| PBS, ischemia | 0.8933600137796899 |
| A-beta, sham | 0.9617181487986436 |
| A-beta, ischemia | 1.0623282231829536 |collagen IV
VEGF-A
Relative mRNA Expression
(fold change)
–
+
–
+
BCCAO
PBS
Ab
–
+
–
+
BCCAO
PBS
Ab
Supplemental Figure 3. Effect of BCCAO with/without Aβ1-40 on cerebrovascular function markers. mRNA expression of collagen IV
 and VEGF-A in the hippocampus 3 weeks after injection by real-time RT-PCR in WT and smAT2 mice are shown.
 *P<0.05, †P<0.01 vs. PBS, BCCAO (-). n=6 for each group in WT and smAT2, respectively.
